# Secondary Metabolites of *Biscogniauxia*: Distribution, Chemical Diversity, Bioactivity, and Implications of the Occurrence

**DOI:** 10.3390/toxins15120686

**Published:** 2023-12-06

**Authors:** Sari Purbaya, Desi Harneti, Wahyu Safriansyah, Rahmawati, Asri Peni Wulandari, Yeni Mulyani, Unang Supratman

**Affiliations:** 1 Department of Chemistry, Faculty of Science and Informatics, Universitas Jenderal Achmad Yani, Cimahi 40531, Indonesia; saripurbaya@gmail.com; 2 Departments of Chemistry, Faculty of Mathematics and Natural Sciences, Universitas Padjadjaran, Jatinangor 45363, Indonesia; desi.harneti@unpad.ac.id (D.H.); wahyu17002@mail.unpad.ac.id (W.S.); yeni.mulyani@unpad.ac.id (Y.M.); 3 Central Laboratory, Universitas Padjadjaran, Jatinangor 45363, Indonesia; rahmawati@unpad.ac.id; 4 Department of Biology, Faculty of Mathematics and Natural Sciences, Universitas Padjadjaran, Jatinangor 45363, Indonesia; asri.peni@unpad.ac.id

**Keywords:** endophytic fungi, *Biscogniauxia*, biological activity

## Abstract

The genus *Biscogniauxia*, a member of the family Xylariaceae, is distributed worldwide with more than 50 recognized taxa. *Biscogniauxia* species is known as a plant pathogen, typically acting as a parasite on tree bark, although certain members of this genus also function as endophytic microorganisms. *Biscogniauxia* endophytic strain has received attention in many cases, which includes constituent research leading to the discovery of various bioactive secondary metabolites. Currently, there are a total of 115 chemical compounds belonging to the class of secondary metabolites, and among these compounds, fatty acids have been identified. In addition, the strong pharmacological agents of this genus are (3a*S*,4a*R*,8a*S*,9a*R*)-3a-hydroxy-8a-methyl-3,5-dimethylenedecahydronaphto [2,3-*b*]furan-2(3*H*)-one (HDFO) (antifungal), biscopyran (phytotoxic activity), reticulol (antioxidant), biscogniazaphilone A and B (antimycobacterial), and biscogniauxone (Enzyme GSK3 inhibitor). This comprehensive research contributes significantly to the potential discovery of novel drugs produced by *Biscogniauxia* and holds promise for future development. Importantly, it represents the first-ever review of natural products originating from the *Biscogniauxia* genus.

## 1. Introduction

A significant variety of natural products have been isolated and identified from many fungi, consisting of approximately 47% of the roughly 33,500 bioactive microbial metabolites [1]. Endophytic fungi are organisms that live in plant tissues without causing harm and produce biologically active compounds specific to the host plants [2]. They are considered a potential source of new bioactive natural material for new drug development, inhabiting the inner tissues of living plants [3,4]. Some endophytic fungi can produce the same or similar bioactive substances as those found in the host plant, one of which is the genus *Biscogniauxia* [5].

*Biscogniauxia* is a genus of fungi in the family Xylariaceae with more than 50 recognized taxa worldwide [6,7]. *Biscogniauxia* endophytic strain has received attention in many cases, which includes constituent research leading to the discovery of various bioactive secondary metabolites [4]. Since the first report on the secondary metabolites of *Biscogniauxia* in 2005, a large number of chemical compounds have been isolated [8,9]. Subsequently, an extensive literature review was carried out using databases from SCI-Finder, Google Scholar, Web of Science, Scopus, Science Direct, PubMed, Chemical Abstracts, ACS journals, Springer, Taylor Francis, Bentham Science, and IOP Science. This comprehensive search yielded numerous articles providing an overview of compounds, including secondary metabolites and fatty acids, from approximately nine taxa of *Biscogniauxia*.

Chromatographic and spectroscopic methods such as nuclear magnetic resonance (NMR), ultraviolet-visible (UV-Vis), infrared (IR), optical rotation (OR), circular dichroism (CD), and mass (MS) explanation of spectroscopy were used in the latest review to elucidate the structures of the 115 isolated compounds. These compounds belong to diverse classes, including azaphilone derivatives, bergamotenes, cerebrosides, coumarins, fatty acids, flavonoids, furan, guaianoids, hydroxycinnamic acids and derivatives, lignans, naphthoquinones, peptides, phenyl and phenol derivatives, phthalide, pyranopyran, α-pyrones, terpenoids and the derivates, tyramines, and others. The composition of the isolated compounds is arranged alphabetically according to their names. In addition, *Biscogniauxia* exhibits various bioactivities, such as antifungal, antimycobacterial, antiproliferative, antioxidant, anticancer, anti-germinative, inhibition of GSK-3β enzyme activity, phytotoxic activity, and AcHE activity, and its potential applications.

## 2. Secondary Metabolites

*Biscogniauxia* produces a large number of low-molecular-weight compounds with native structure and bioactivity. In this situation, these fungi produce metabolites with enormous structural diversity that belong to the various classes of natural products presented in Table 1.

### 2.1. Azaphilone Derivatives

**Figure 1:**
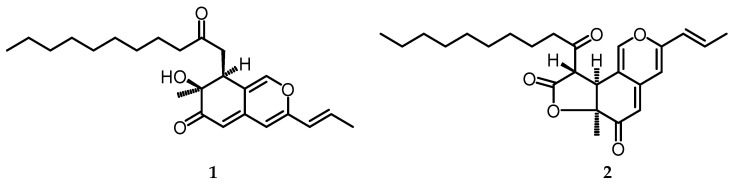
Structures of azaphilone derivatives Biscogniazaphilones A (**1**) and Biscogniazaphilones B (**2**).

A new azaphilone derivative was successfully isolated from the *n*-BuOH soluble fraction of 95% EtOH extract from substrate culture on *B. formosana* to produce Biscogniazaphilones A (**1**) and Biscogniazaphilones B (**2**) [10] (Figure 1). This marks the first report of this compound being isolated from *B. formosana*. Subsequently, azaphilones or azaphilonoids are fungal polyketides known for the highly oxygenated cyclic pyranoquinone core, usually referred to as isochromenes and quaternary carbon centers, and also known as pigments [24,25]. Compounds 1 and 2 based on the spectra of ^1^H NMR and ^13^C NMR are similar, but compound 2 has one γ-lactone group between C-6a and C-9.

### 2.2. Cerebrosides

**Figure 2:**
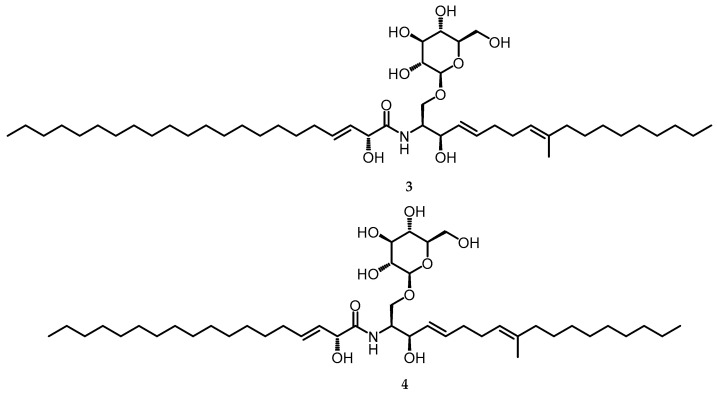
Cerebroside A (**3**) and Cerebroside C (**4**).

Cerebrosides are a family of glycosphingolipids and important components of various tissues and organs in biological systems. Chemically, cerebrosides consist of hexose and ceramide groups, typically consisting of long-chain amino alcohols commonly called “sphingoid bases” (=sphingosine or sphingol) and amide-linked long-chain fatty acids [26]. While cerebrosides can be found in plants, fungi, and animals, distinct variations exist in the structure of the ceramide backbone among these organisms [27]. In the genus *Biscogniauxia*, Cerebroside A (**3**) and Cerebroside C (**4**) (Figure 2) were successfully isolated from the ethyl acetate extract in the stroma of *Biscogniauxia whalleyi* mushrooms cultivated on potato dextrose agar (PDA) media. Fungal cerebrosides exhibited remarkable structural conservation, with modifications including different unsaturation sites as well as varying lengths of fatty acid residues in the ceramide moiety [28]. 

### 2.3. Coumarin

**Figure 3:**
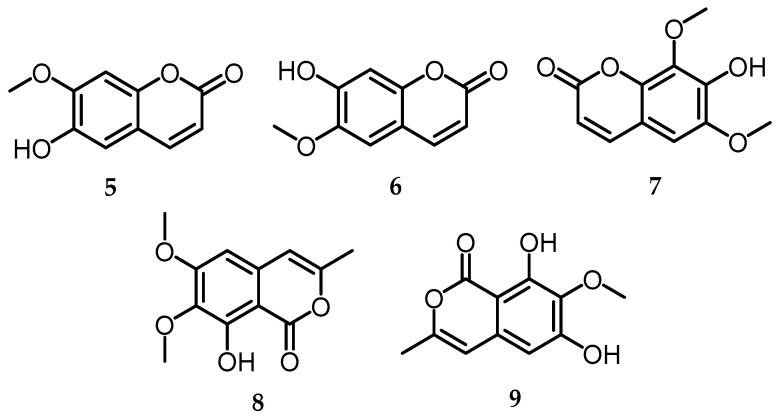
Structures of coumarin and isocoumarin (**5**–**9**).

Coumarin is a secondary metabolite derived from 1,2 benzopyrone, formed from the benzene ring, and α-pyrone found in microorganisms and higher plants, originating from the phenylpropanoid pathway [29,30]. Coumarin has been extensively examined as one of the most promising structures for the development of new agents with higher specificity and affinity against molecular targets. Furthermore, it is characterized by its intrinsic properties such as antimicrobial, antioxidant, anti-inflammatory, antiadipogenic, cytotoxic, apoptotic, antiproliferative, antimycobacterial activity against *Mycobacterium tuberculosis*, antileishmanial, antiviral, anticancer, and cytotoxic agent [31]. *Biscogniauxia* endophytic strain has garnered attention in constituent research, leading to the discovery of various bioactive secondary metabolites, specifically in the context of coumarins. Given their wide range of pharmacological values, coumarins and their derivatives hold significant importance in synthesis and production. The coumarin is produced from *Biscogniauxia* and is divided into three groups: coumarin compounds **5**–**7**, isocoumarins **8**–**9**, and dihydroisocoumarin (Melleins) compounds **10**–**25** (Figure 3).

#### 2.3.1. Coumarin

Isoscopoletin (**5**), scopoletin (**6**), and isofraxidin (**7**) were successfully isolated from the *n*-BuOH-soluble endophytic fungus *Biscogniauxia cylindrospora* [4] (Figure 3). Regarding scopoletin (**6**), before it was successfully isolated from *B. cylindrospora*, it had been widely produced from plants and could be isolated from various plant parts (roots, fruit, leaves, stems, etc.) such as *Nicotiana tabacum*, *Sinomenium acutum*, *Helichrysum italicum*, *Manihot esculenta*, *Aegle marmelos*, *Chenopodiastrum murale*, *Hypochaeris radicata*, *Cirsium setidens*, *Aleurites moluccana* L., *Morinda citrifolia*, *Ipomoea digitata* L., *Ipomea reniformis*, A*rtemisia iwayomogi*, *Macaranga gigantifolia* M., *Artemisia annua*, *Tetrapleura tetraptera*, *Tilia cordata* M., *Melia azedarach* L., *Acer saccharum* M., *Hymenodictyon obovatum*, *Fagraea ceilanica*, and *Morus alba* L. [32].

#### 2.3.2. Isocoumarin

Chromatographic separation of the EtOAc extract from cultured *Biscogniauxia capnodes* yielded two isocoumarins, 6-*O*-methyl-reticulol (**8**) and reticulol (**9**). 6-*O*-methyl-reticulol was previously isolated from *Streptomyces mobaraensis* with inhibitory activity in the liverwort *Wettsteinia ÿschusterana* [33](Figure 3).

#### 2.3.3. Dihydroisocoumarin (Melleins)

**Figure 4:**
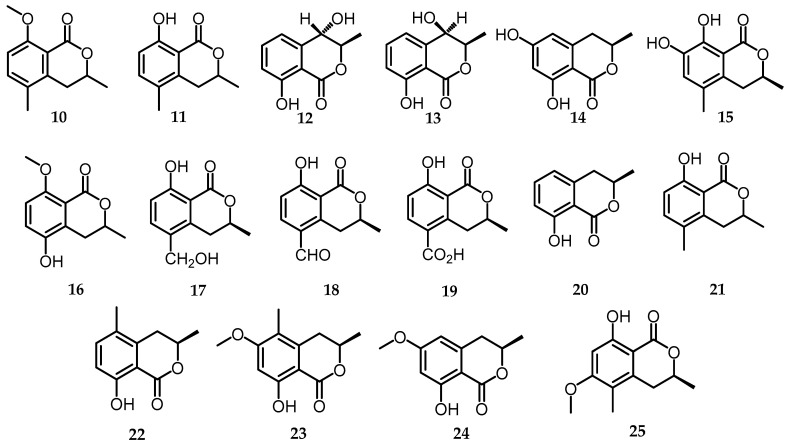
Melleins structures (**10**–**25**).

Mellein is a secondary metabolite of the 3,4-dihydroisocoumarin subgroup, which is a structural isomer of coumarin and belongs to the polyketide class. It is abundant in microorganisms and higher plants and has many biological activities [34]. The content of mellein in *Biscogniauxia* is higher than that of isocoumarins; namely, 16 compounds were successfully isolated, as shown in Figure 4. One new dihydroisocoumarin (3*S*)-5-hydroxy-8-*O*-methylmellein (**16**), along with the other 3 mellein compounds 5-hydroxymethylmellein (**17**), 5-formylmellein (**18**), and mellein-5-carboxylic acid (**19**), was isolated from the *n*-BuOH soluble fraction of 70% EtOH extract of rice fermented with the endophytic fungus *B. cylindrospora* (BCRC 33717) [15]. Mellein compounds have also been isolated from *B. rosacearum* oak strain IRAN 4194C and are important for producer organisms and included in many biological activities, including phytotoxicity. Several related melleins are (3*R*)-mellein (**20**) and (3*R*,4*R*)-and (3*R*,4*S*)-4-hydroxymellein (**12**) and (**13**), (3*R*)-6-hydroxymellein (**14**), and (3*R*)-4-methoxymellein (**24**) were also isolated from *B. rosacearum* IRAN 4287C [14]. Two mellein derivatives, namely 3,5-dimethyl-8-methoxy-3,4-dihydroisocoumarin (**10**) and 3,5-dimethyl-8-hydroxy-3,4-dihydroisocoumarin (**11**), were isolated from *Biscogniauxia nummularia*. Furthermoe, in *Biscogniauxia mediterranea* extract strain LF657, which was isolated from marine sediments east of the Mediterranean Sea, a mellein derivative was identified as 6-methoxy-5-methylmellein (**25**) [17]. Additionally, *B. capnodes* yielded 7-hydroxy-5-methylmellein (**15**), and 5-methylmellein (**21**) was isolated from various sources, including *B. mediterranea* (EtOAc), *B. mediterranea* (DCM), *B. capnodes* (EtOAc), and *B. whalleyi* (EtOAc) [11].

### 2.4. Fatty Acids

**Figure 5:**
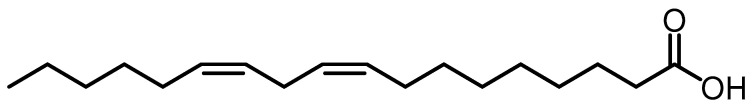
Linoleic acid (**26**).

From the *Biscogniauxia* endophytic strain, one fatty acid that was effectively isolated was linoleic acid (**26**), obtained in the form of a colorless oil. This isolation was achieved from the EtOAc-soluble fraction of a 95% EtOH rice extract that underwent fermentation with *B. cylindrospora* [4] (Figure 5).

### 2.5. Flavonoids

**Figure 6:**
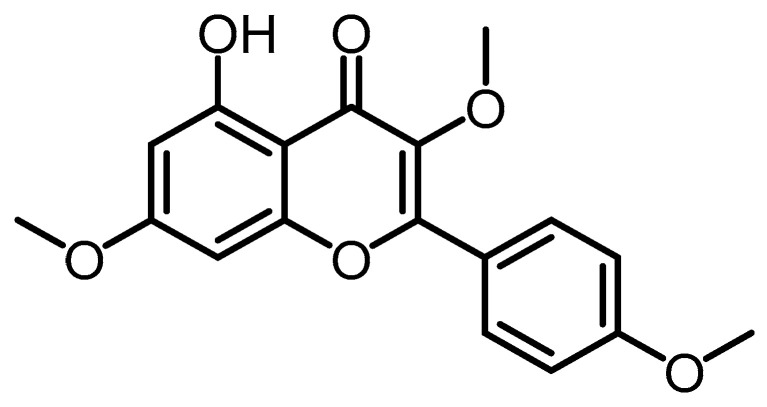
5-hydroxy-3,7,4′-trimethoxyflavone (**27**).

One flavonoid was further isolated from the *n*-BuOH-soluble fraction of the 95% EtOH extract from the solid substrate culture of *B. formosana* BCRC 33718, namely 5-hydroxy-3,7,4′-trimethoxyflavone (**27**) [10], which had previously been isolated from the stem wood and bark of *Aniba* species [35] (Figure 6).

### 2.6. Furan

**Figure 7:**
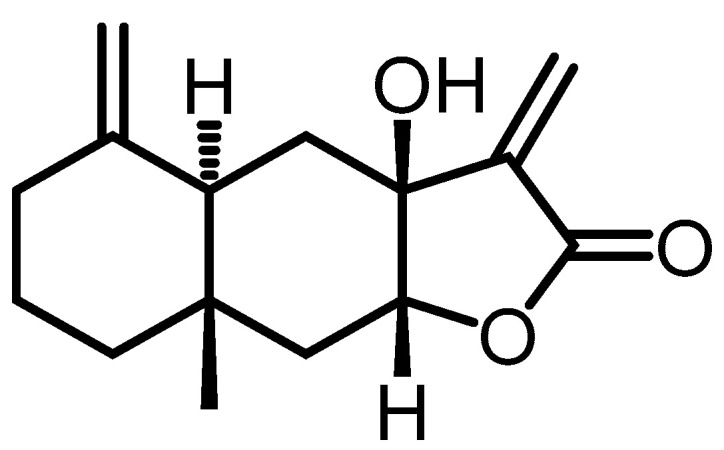
(3a*S*,4a*R*,8a*S*,9a*R*)-3a-hydroxy-8a-methyl-3,5-dimethylenedecahydronaphto [2,3-*b*]furan-2(3*H*)-one (HDFO) (**28**).

(3a*S*,4a*R*,8a*S*,9a*R*)-3a-hydroxy-8a-methyl-3,5-dimethylenedecahydronaphto [2,3-*b*]furan-2(3*H*)-one (HDFO) (**28**) isolate from *Biscogniauxia* sp. was used as a control for the detection of the growth inhibition zone O-811 ECF inhibitor compound against *M. oryzae*. The customized HDFO of O-821-ECF showed inhibitory activity against *M. oryzae* at <5 ppm [18] (Figure 7).

### 2.7. Hydroxycinnamic Acids and Derivatives

**Figure 8:**
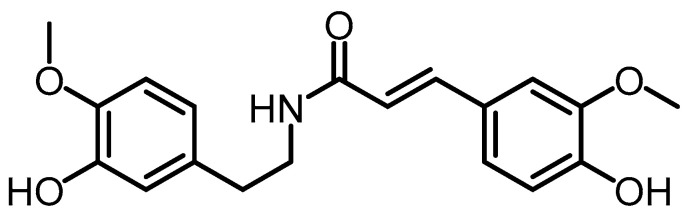
Structure of *N*-*trans*-feruloy-3-*O*-methyl-dopamine (**29**).

In the category of hydroxycinnamic acids and derivatives, after a series of isolation stages, dried rice from *B. formosana* BCRC 33718 was extracted with 95% EtOH and subjected to preparative TLC using MeOH as a developer. This process yielded *N*-*trans*-feruloy-3-*O*-methyl-dopamine (29), the sole compound in the hydroxycinnamic acids and derivatives group. This compound had previously been identified in spinach leaves (*Spinacia oleracea*) [36] (Figure 8).

### 2.8. Lignans

**Figure 9:**
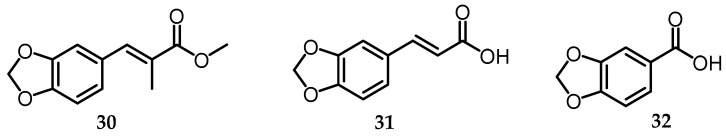
Structure of lignan (**30**–**32**).

Lignans, which are an abundant class of phenylpropanoids, have received wide attention in many fields. This is mainly because these compounds have some medically important biological activities, for example antitumor, antimitotic, and antiviral properties [37]. Three lignans were successfully isolated from the *n*-BuOH soluble fraction of 95% EtOH extract from *B. formosana* solid substrate culture, including methyl 3,4-methylenedioxycinnamate (**30**), 3,4-methylenedioxycinnamic acid (**31**), and 3,4-methylenedioxybenzoic acid (**32**) [10] (Figure 9).

### 2.9. Naphthoquinones

**Figure 10:**
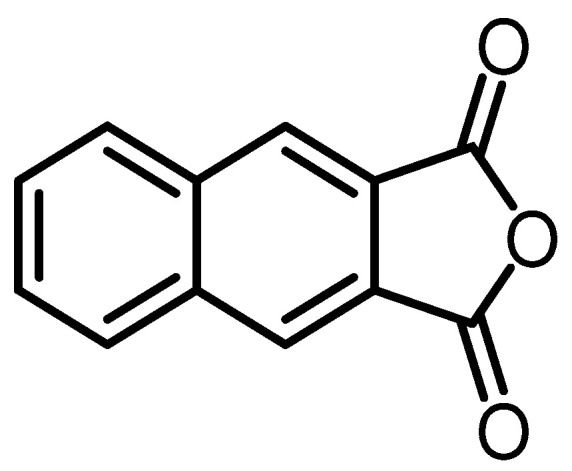
Structure of naphtho [2,3-c]furandione (isofuranonaphthoquinone) (**33**).

Naphthoquinones are widespread and have been found in higher plants, fungi, and actinomycetes [38]. In the extract of *B. mediterranea* strain LF657, isolated from deep-sea sediments in the eastern Mediterranean Sea at a water depth of 2800 m, the new isopyrrolonaphthoquinone compound naphtho [2,3]furandione (isofuranonaphthoquinone) (biscogniauxone) (**33**) was identified [17] (Figure 10). Many researchers have an interest in this class of naphthoquinone compounds because of their wide range of biological activities, such as phytotoxic, insecticidal, antibacterial, and fungicide. In addition, some of these compounds also have cytostatic [39] and anticarcinogenic properties [38]. As for compound (**33**), it shows inhibitory activity against the GSK-3β enzyme [17], which will be explained further in Section 3.

### 2.10. Peptides

**Figure 11:**
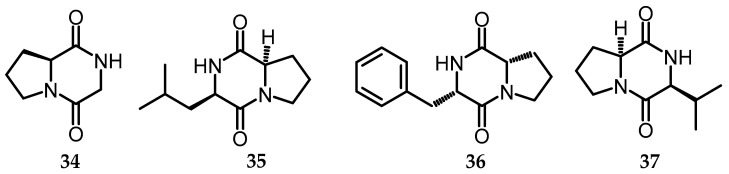
Structures of cyclic dipeptides (**34**–**37**).

**Figure 12:**
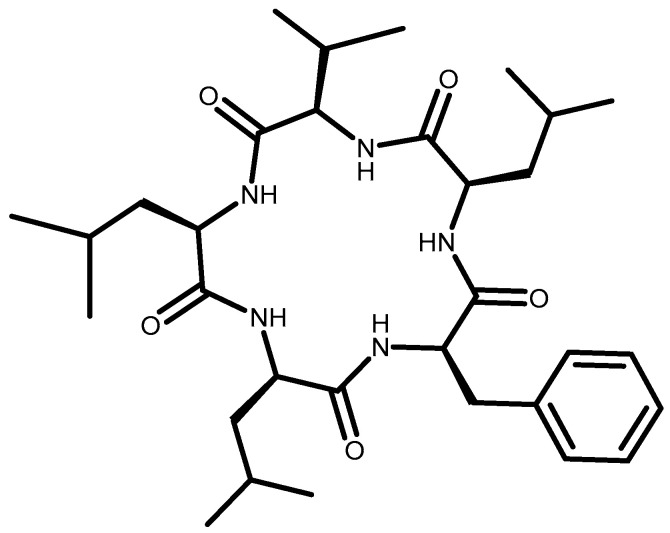
Structure of cyclo-(ʟ-Phe-ʟ-Leu-ʟ-Val-ʟ-Leu-ʟ-Leu) (**38**).

In the last 20 years, the field of peptides has witnessed major developments, stimulated by the discovery of some bioactive peptides [40], one of which is in fungi. A total of 1133 peptides with antifungal properties have been reported in the Antimicrobial Peptide Database (APD) [41]. The chemical constituents of *B. whalleyi* (Graph ostomataceae) strain SWUF13-085 were isolated using chromatography techniques, which resulted in the isolation of 35 compounds, one of which was in the peptide group, including cyclic dipeptides in compounds **33**–**36** [11]. Cyclic dipeptides, known as diketopiperazines (DKP), the simplest cyclic forms of peptides, are widespread and unrivaled in their structural and biofunctional diversity. Subsequently, *B. whalleyi* successfully isolated include cyclo (l-Pro-Gly) (**34**), cyclo (ʟ-Pro-ʟ-Leu) (**35**), cyclo (ʟ-Pro-ʟ-Phe) (**36**), and cyclo (ʟ-Pro-ʟ-Val) (**37**) (Figure 11). Besides the use of Preparative HPLC (C18) cyclopeptide, cyclo-(ʟ-Phe-ʟ-Leu-ʟ-Val-ʟ-Leu-ʟ-Leu) (**38**) (Figure 12) from *B. mediterranea* was a previously synthesized derivative of the fungal metabolite sansalvamide A [42,43].

### 2.11. Phenyl and Phenol Derivatives

**Figure 13:**
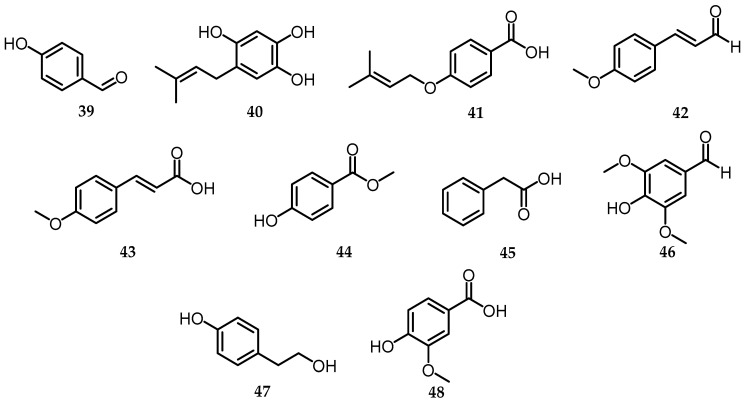
Structures of phenyl and phenol derivatives (**39**–**48**).

Four prenyl and phenol derivative compounds were isolated from solid substrate cultures of *B. formosana* BCRC 33718, including 4-hydroxybenzaldehyde (**39**), 4-(3-methylbut-2-enyloxy)benzoic acid (**41**), 4-methoxycinnamaldehyde (**42**), and 4-methoxy-*trans*-cinnamic acid (**43**) (Figure 13). Meanwhile, from the *n*-BuOH soluble fraction of the 70% EtOH extract of *B. cylindrospora*, 3 compounds were successfully isolated, namely methylparaben (**44**), syringaldehyde (**46**), and vanillic acid (**48**) (Figure 13). Phenylacetic acid (**45**) (Figure 13), obtained by EtOAc extraction from the culture filtrate (10 L) of *B. mediterranea*, was fractionated using a combination of column chromatography and thin layer chromatography to identify the most polar metabolites [8]. Furthermore, the chemical constituents of *B. whalleyi,* namely 5-hydroxy-2-prenylhydroquinone (**40**) and tyrosol (**47**), are metabolites that are commonly produced by plants and microorganisms through the shikimate biosynthetic pathway [44] and are also produced by many fungal species [45] (Figure 13). Tyrosol was also produced from the *B. rosacearum* oak strain, which had phytotoxic activity showing severe necrosis at the highest concentrations when tested on oak leaves (*Quercus ilex* L.) [14].

### 2.12. Phthalides

**Figure 14:**
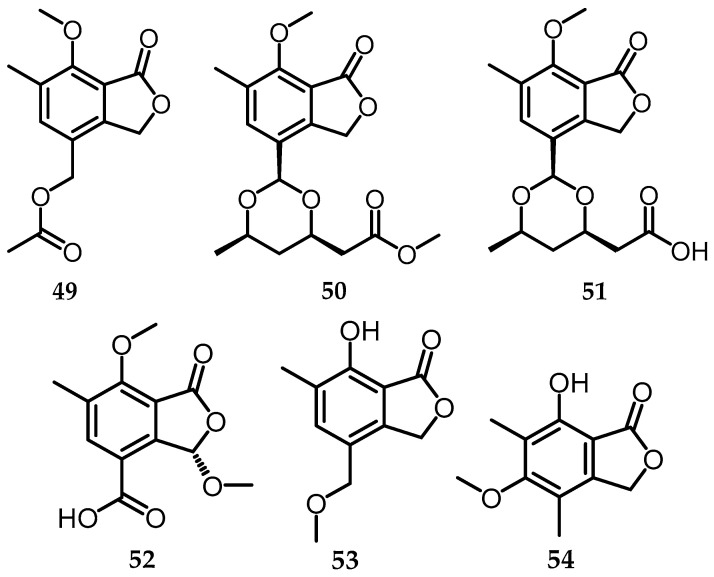
Structure of phthalide (**49**–**54**).

A new phthalide derivative known as biscogniphthalides A–D (**50**–**53**)was successfully isolated from *Biscogniauxia* sp. (No. 69-8-7-1) along with one known related phthalide, [4-[(acetyloxy)methyl]-7-methoxy-6-methyl-1(3*H*)-isobenzofuranone (**49**) [9] (Figure 14). One phthalide was also isolated from *B. whalleyi*, namely 7-hydroxy-5-methoxy-4,6-dimethylphthalide (**54**) [11] (Figure 14). These phthalides are isobenzofuranones known as 3H-isobenzofuran-1-one, characterized by a bicyclic core originating from the fusion of γ-lactone (ring A) with benzene (ring B) [46]. Furthermore, phthalide is widely found in plants, fungi, and liverworts and exhibits various interesting biological activities, such as antimicrobial, neuroprotection, anti-anginal, anti-platelet aggregation, anti-smooth muscle proliferation, anti-thrombosis, cardiac function, modulation, and protection against cerebral ischemia [46,47,48]. Compounds **48**–**52** were tested for anti-acetylcholinesterase (AChE), anti-microbial, and anti-α-glucosidase activities, which will be explained further in Section 3.

### 2.13. Pyranopyran

**Figure 15:**
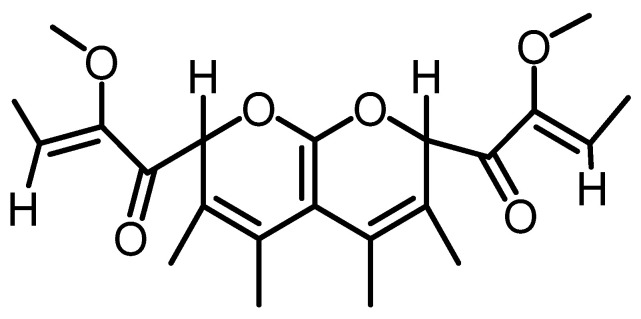
Structure of biscopyran (**55**).

Biscopyran **55** is a resubstituted pyranopyran identified using the spectroscopic methods as (*Z*)-2-methoxy-1-[7-((*Z*)-2-methoxybut-2-enoyl)-3,4,5,6-tetramethyl,2*H*,7*H*-pyrano [2,3-*b*]pyran-2-yl]but-2-en-1-one. This compound was isolated from liquid culture filtrate of *B. mediterranea* from infected cork oak (*Q. suber*) stems collected in Sardinia (Italy) with phytotoxic activity [8] (Figure 15).

### 2.14. α-Pyrones

**Figure 16:**
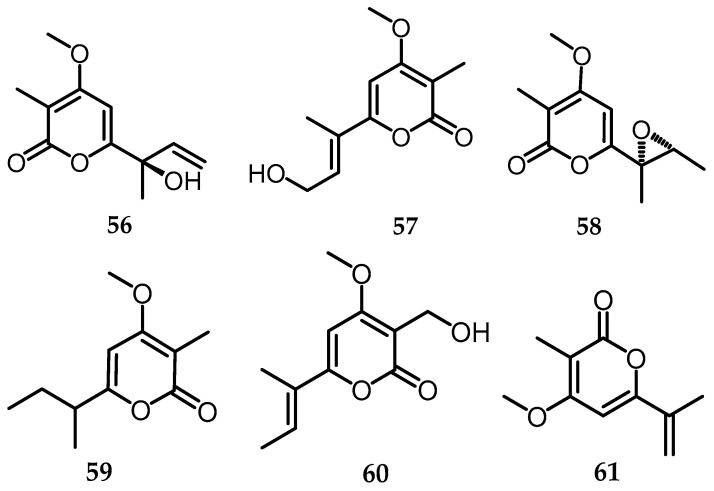
Structures of α-pyrones (**56**–**61**).

*α*-Pyrones (1, also 2-pyrones) are six-membered cyclic unsaturated esters that share chemical and physical properties reminiscent of alkenes and aromatic compounds. These compounds are abundant in bacteria, microbial systems, plants, insects, and animals [49]. Regarding the α-pyrones derivatives, one of them is *B. whalleyi* SWUF13-085, distinguishable through the NMR spectra, with data compared to existing literature [11]. Additionally, 7 compounds were successfully isolated, including 6-(1′,2′-dimethyloxiran-1′-yl)-4-methoxy-3-methyl-2*H*-pyran-2-one (**56**) [50], gulypyrone B (**57**) [51], 6-[(1*R*)-1-hydroxy-1-methyl-2-propenyl]-4-methoxy-3-methyl-2*H*-pyran-2-one (**58**) [52], nectriapyrone (**59**) [51], phomopyrone A (**60**) [53], and vermopyrone (**61**) [54] (Figure 16). Nectriapyrone (**59**), also isolated from *B. rosacearum* oak strain IRAN 4287C tested on the grapevine, showed the presence of severe necrosis at the highest concentrations. Besides, nectriapyrone (**59**) was previously isolated as a phytotoxin produced by a phytopathogenic fungus such as *Diaporthe angelicae* (anamorph *Phomopsis foeniculi*), which is the causative agent of fennel disease (*Foeniculum vulgare*) in Bulgaria [55].

### 2.15. Steroids

**Figure 17:**
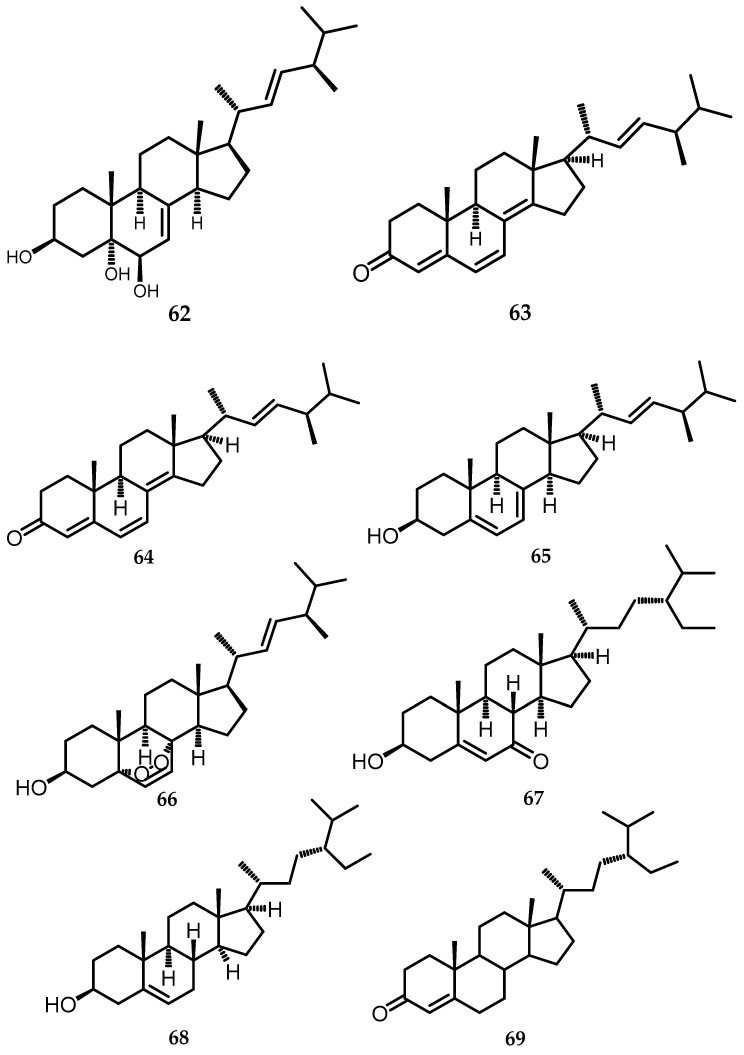
Structures of *steroid*, (**62**–**69**).

Steroids play a crucial role in the active ingredients of medicines found across the animal and plant kingdoms, characterized by a common chemical framework of four fused rings, including three six-membered rings and a five-membered ring [56]. Several steroids are produced from the genus *Biscogniauxia*, including *B. whalleyi*, *B. formosana,* and *B. cylindrospora*. In the *n*-BuOH soluble fraction, a 95% EtOH extract of long grain rice produced by the endophytic fungus *B. formosana BCRC 33718* was fractionated with a combination of silica gel, RP-18 column, and preparative TLC to produce 12 compounds, and one of them was a steroid group, namely ergosta-4,6,8(14),22-tetraen-3-one (**64**) [10], which was previously isolated from *Ganoderma applanatum* [57]. Furthermore, 4 steroid compounds were also isolated from *B. whalleyi* based on data from intensive comparisons of NMR data, specific optical rotation values, and MS data with those in the literature, indicating that the isolated compound were cerevisterol (**62**) [58], ergone (**63**) [59], ergosterol (**65**) [58], and ergosterol peroxide (**66**) [60] (Figure 17). Three steroid compounds were also isolated from *B. cylindrospora,* namely 3β-hydroxystigmast-5-en-7-one (**67**), which was produced in rice from *B. cylindrospora* BCRC 33717, then extracted three times with 70% EtOH at room temperature [15] and two steroids, β-sitostenone (**68**) and β-sitosterol (**69**), in the EtOAc soluble fraction of *B. cylindrospora* BCRC 33717 [4] (Figure 17).

### 2.16. Terpenoids and Their Derivatives

#### 2.16.1. Diterpenoids

**Figure 18:**
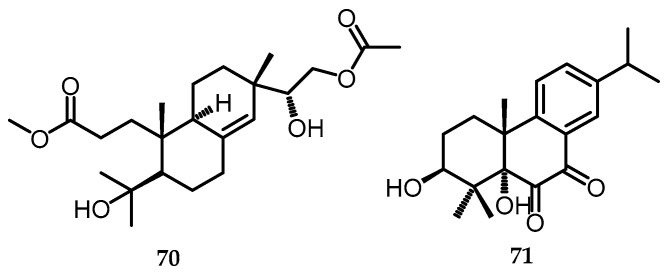
Structures of diterpenoids (**70**) and (**71**).

Two new diterpenoids, namely biscognisecoisopimarate A (**70**), including the seco-isopimarane type, and 3β-hydroxyrickitin A (**71**), the abietane type, were obtained from *Biscogniauxia* sp. (No. 71-10-1-1) [19]. Subsequently, diterpenoids are natural compounds with a C-20 carbon skeleton that are chemically heterogeneous. They are widely distributed from the condensation of four isoprene units derived from the mevalonate or deoxysylulose phosphate pathway. Diterpenoids can be classified as linear, bicyclic, tricyclic, tetracyclic, pentacyclic, or macrocyclic diterpenes, depending on their core framework. In nature, they are generally found in polyoxygenated forms with keto and hydroxyl groups, which are often esterified by small aliphatic or aromatic acids [61]. Biscognisecoisopimarate A (**70**) was obtained as colorless needle crystals, while 3β-hydroxyrickitin A (**71**) (Figure 18) was obtained as a yellowish oil, which was then tested for its biological activity for anti-Alzheimer disease (AD) activity, anti-inflammatory, and cytotoxic activity [20], which will be discussed further in Section 2.

#### 2.16.2. Meroterpenoid

**Figure 19:**
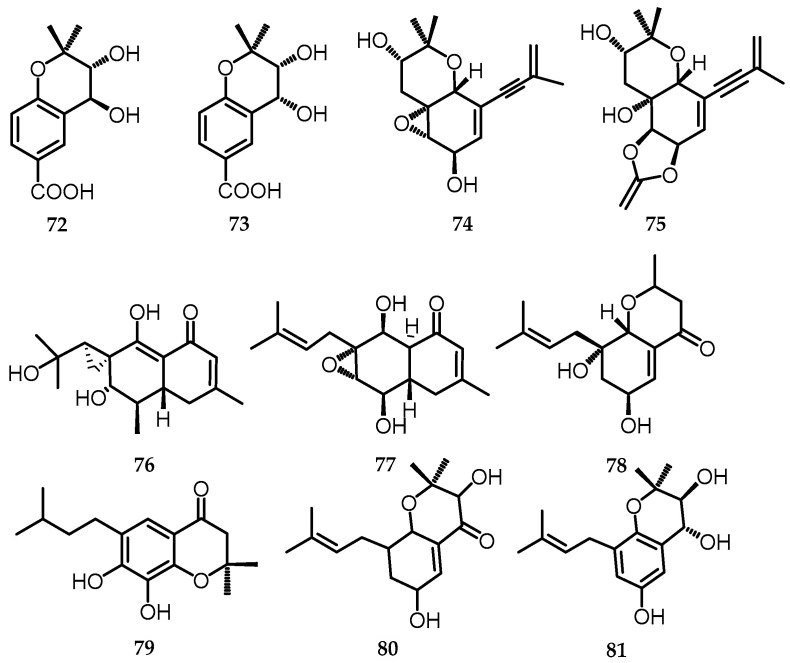
Structures of meroterpenoids (**72**–**81**).

Terpenoids (terpenes and oxygenated derivatives) constitute one of the largest groups of naturally occurring compounds, which are characterized by their very wide distribution in both the plant and animal kingdoms and have also recently been obtained from endophytic fungi [62,63,64]. A terpenoid derivative, namely meroterpenoid, is a compound that is formed from a combination of frameworks between terpenoid and polyketide frameworks, alkaloids, phenols, and amino acids [65,66,67,68]. It can be obtained by bacteria, algae, plants, and animals, as well as endophytic fungi, one of the endophytic fungi producing meroterpenoid compounds, namely *Biscogniauxia* sp. compounds **72**–**81** [20]. Biscogniacid B (**72**), biscogniacid C (**73**), and biscognienyne D (**74**) were obtained as colorless needles, while biscognienyne F (**75**) was obtained as a yellowish oil. Biscognin (A) (**76**) was obtained as colorless needles having a unique 2-isopropyl-6′-methyloctahydro-1′H-spiro[cyclopropane-1,2′-naphthalene] skeleton. Biscognin B (**77**) and E (**80**) were obtained as colorless needles, whereas biscognin C, D, and F (**78**, **79**, and **81**) were all obtained as colorless oils [20] (Figure 19).

**Figure 20:**
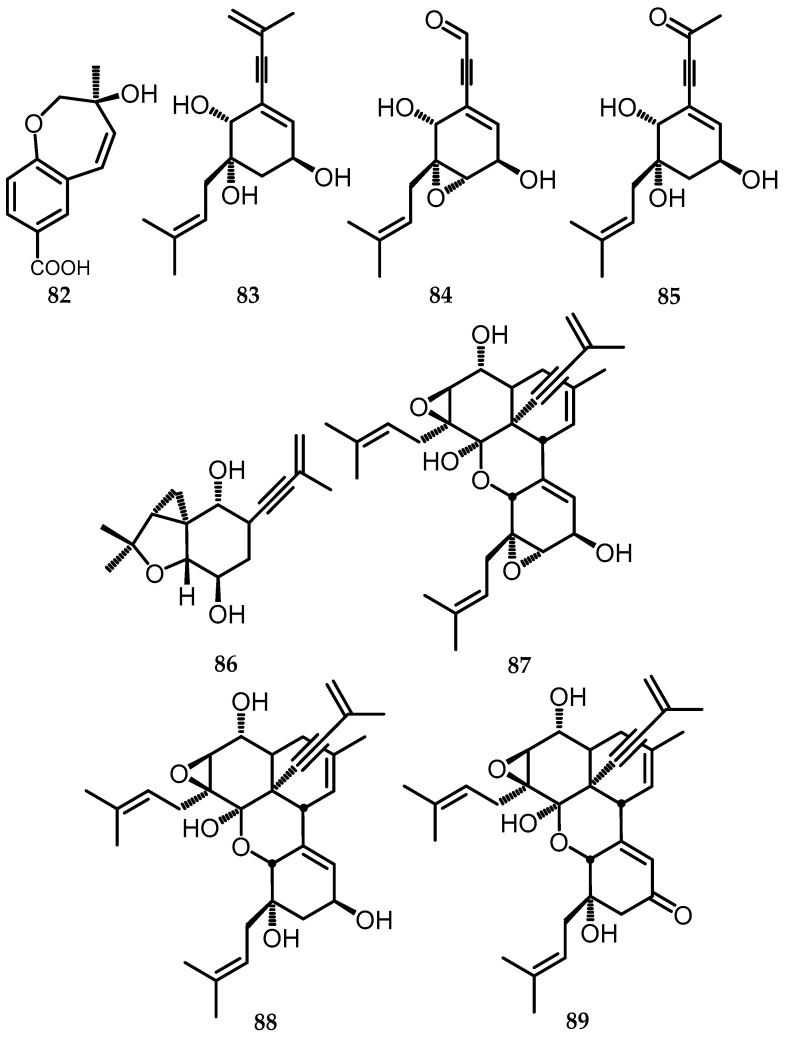
Meroterpenoid structures (**82**–**89**).

A new framework of dimericbiscognienynes A (**87**), namely dimer meroterpenoid type di isoprenyl-cyclohexene, along with three new monomers, including meroterpenoid type diisoprenyl-cyclohexene (biscognienyne A–C, **83**–**85**) and one iso prenyl-benzoic meroterpenoid type biscogniacid acid A (**82**) successfully obtained from *Biscogniauxia* sp. (No. 71-10-1-1), were isolated from the lichen *Usnea mutabilis* Stirt [21]. Additionally, meroterpenoid biscognienyne E (**86**) was obtained via a different fermentation process, increasing the number of fermentations to 60 Erlenmeyer flasks and extending the fermentation time to 50 days [20]. Two new types of diisoprenyl-cyclohexene were also obtained from *Biscogniauxia* sp. (No. 71-10-1-1), namely dimericbiscognienynes B and C (**88** and **89**), adding to the rare class of meroterpenoids [22] (Figure 20).

#### 2.16.3. Sesquiterpenoids

**Figure 21:**
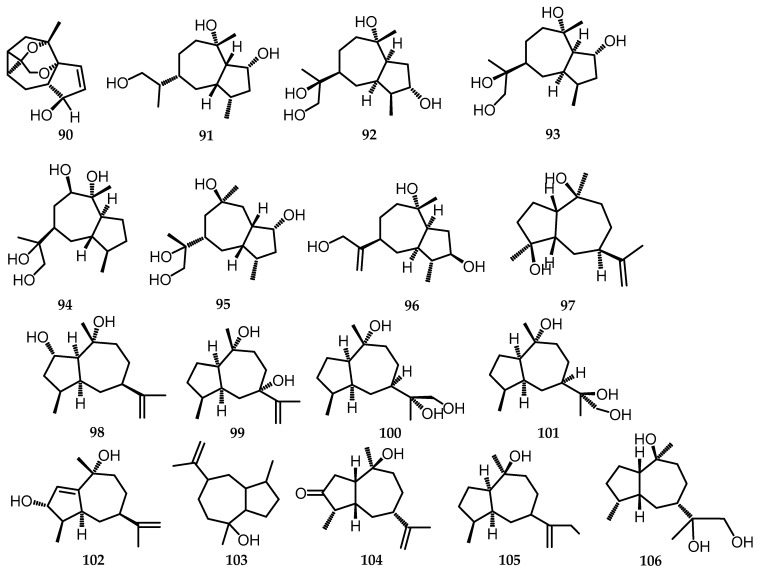
Structures of sesquiterpenoids (**90**–**106**).

Sesquiterpenoids are derived from three isoprene units and exist in various forms, including linear, monocyclic, bicyclic, and tricyclic frameworks. They are the most diverse group of terpenoids [63]. Seven new sesquiterpenoids of the guanine type, namely biscogniauxiaol A–G (**90**–**96**), were successfully isolated from the endophytic fungus *Biscogniauxia petrensis* MFLUCC 14-0151 on *Dendrobium orchids* [23]. The isolated biscogniauxiaol A–G (**90**–**96**) (Figure 21) is a colorless solid that was then tested for its biological activity for antifungal, anti-inflammatory, and anti-cancer activities, which will be explained further in Section 2. Furthermore, 9 guaiane-type sesquiterpenoids were isolated from *B. whalleyi* and immediately recognized from the frequently found NMR spectroscopic pattern (compounds **97**–**105**) (Figure 21) in the entire fungal family *Graphostomataceae* [13,69]. One undescribed compound is (1R*,4S*,5S*,7S*,10R*)-guaia-11(12)-en-7,10-diol (**99**), along with 8 known compounds, including epi-guaidiol A (**97**) [70], graphostromane E (**98**) [71], (1*R*,4*S*,5*S*,7*R*,10*R*,11*R*)-guaiane-10,11,12-triol (**100**) [72], (1*R*,4*S*,5*S*,7*R*,10*R*,11*S*)-guaiane-10,11,12-triol (**101**) [72], patchouliguaiol A (**102**) [73], pogostol (**103**) [74], xylaranone (**104**) [13], and xylaranol A (**105**) [74]. Xylaranone (**104**), the sesquiterpene guaiane, which was previously reported together with the terpenoid xylaranol B (**106**), was also isolated from *B. nummularia* [13].

**Figure 22:**
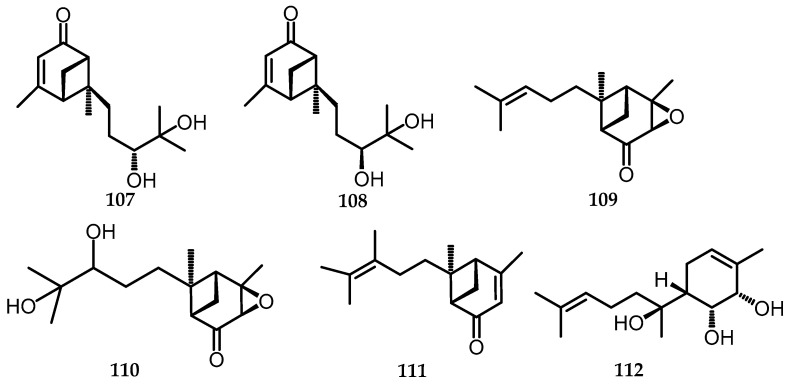
Structures of sesquiterpenoids (**107**–**112**).

Bergamotene, a bicyclic sesquiterpene, is found in plants, insects, and fungi with α-*trans*-bergamotene, which is the most abundant compound [75]. Bergamotene and its related structures (bergamotane sesquiterpenoids) have been shown to have various biological activities such as antioxidant, anti-inflammatory, immunosuppressive, cytotoxic, antimicrobial, antidiabetic, and insecticidal effects [76] Six compounds were isolated from *B. whalleyi* SWUF13-085 using chromatographic techniques, four of which were xylariterpenoid L (**109**), xylariterpenoid M (**110**), xylariterpenoid N (**111**), and (1*R*,2*S*,6*R*,7*S*)-1,2-dihydroxy-α-bisabolol (**112**), which is a newly discovered compound. The remaining two compounds, xylariterpenoids A (**107**) and xylariterpenoids B (**108**), were characterized via extensive comparisons of NMR and ECD data with existing literature [69,76,77] (Figure 22).

### 2.17. Tyramine

**Figure 23:**
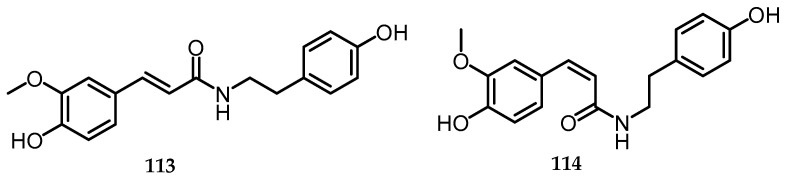
Structures of *N*-*trans*-feruloyltyramine (**113**) and *N*-*cis*-feruloyltyramine (**114**).

Tyramine, a naturally occurring trace amine derived from the amino acid tyrosine, can exist in three isomers: 2-, 3-, or 4-hydroxyphenylethylmaine, which are commonly referred to as *ortho*-, *meta*-, and *para*-tyramine [78]. *N*-*trans*-feruloyltyramine (**113**) and *N*-*cis*-feruloyltyramine (**114**) are tyramines that were isolated from the *n*-BuOH-soluble fraction of 70% EtOH extract of fermented rice with the endophytic fungus *B. cylindrospora* (BCRC 33717) [15] (Figure 23).

### 2.18. Other

**Figure 24:**
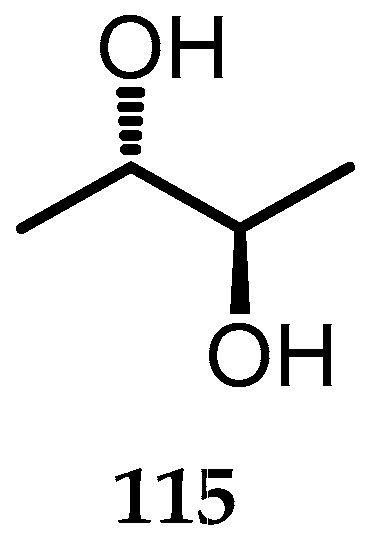
Structure of meso-2,3-butanediol (**115**).

Meso-2,3-butanediol (**115**) is the only phytotoxin isolated from *Biscogniauxia rosacearum* (IRAN 4194C) (Figure 24). This compound was first identified as a pathogen included in GTD in the Paveh vineyard, Kermanshah Province (west of Iran) [14]. This compound showed no optical activity and was identified based on spectroscopic data, as reported by Gallwey et al., 1990 [79].

## 3. Biological Activity

Mushrooms, including various fungal species, are a valuable source of numerous secondary metabolites with diverse chemical structures and a wide range of biological activities [25,80]. Fungi, in general, have well-developed secondary metabolic pathways, and the sheer diversity of fungal species and the biosynthetic gene pools suggest a nearly limitless potential for metabolic variation. This diversity serves as an untapped resource for drug discovery and synthetic biology [1]. Among these fungi, one of the fungal species that is rich in biologically active secondary metabolites is endophytic fungi [81]. They are found on a variety of plant hosts, ranging from herbaceous plants in a variety of habitats, including extreme arctic, alpine, and xeric environments, to subtropical and mesic tropical forests [82]. Subsequently, nearly 300,000 plant species on Earth host one or more endophytics [83], and one such endophytic is the *Biscogniauxia* endophytic strain. Currently, secondary metabolites from *Biscogniauxia* show a variety of biological activities and have become important candidates for the development of new drugs, which are summarized in Table 2.

### 3.1. Antifungal

The 5-methylmellein compound (**21**) showed moderate antifungal activity against phytopathogenic fungi *Phomopsis viticola* and *Phomopsis obscurans* (50%–63% inhibition, respectively). However, it showed weak antifungal activity (up to 20% inhibition) against *B. cinerea*, *C. fragariae*, and *F. oxysporum* at 300 µM. The results were derived from testing endophytic fungal communities associated with the medicinal plant cactus *Opuntia humifusa* (Cactaceae) from the United States, which was isolated from extracts of *B. mediterranea* Ohu 19B [16]. However, apart from *B. mediterranea,* compound **20** was also successfully isolated from the endophytic fungus; *B. capnodes* was isolated from *Averrhoa carambola* fruit [12], and endophytic fungus *B. whalleyi* was resurrected to the Graphostomataceae family [11]. 5-methylmellein is a dihydroisocoumarin known for its antibacterial and antifungal properties against various microorganisms [84,85]. Furthermore, Han et al. 2023 isolated 7 new compounds of the guaiane-type sesquiterpenoid group from the endophytic fungus *B. petrensis* on *Dendrobium orchids* against *Candida albicans* (336485). All compounds exhibited inhibitory activity against *C. albicans*, with biscogniauxiaol A (**90**), biscogniauxiaol B (**91**), and biscogniauxiaol F (**95**) showing strong inhibitory activity with MICs of 1.60, 6.25, and 6.30 µM, respectively (amphotericin B and fluconazole with MICs of 0.43 and 2, respectively; 0.61 µM) [23].

### 3.2. Antimycobacterial

The biological activity of the endophytic fungus *Biscogniauxia formosana* BCRC 33718, originating from the bark of the medicinal plant species *Cinnamomum* sp. was tested in vitro against the antimycobacterial *Mycobacterium tuberculosis* strain H37Rv. Furthermore, 2 newly discovered constituents, biscogniazaphilones A (**1**) and B (**2**), showed the strongest antimycobacterial activity against *M. tuberculosis* strain H37Rv, with MIC values of 5.12 and 2.52 mg/mL, respectively. These values were stronger than the ethambutol used as a positive control, with a MIC value of 6.25 mg/mL. Compound **2**, with one γ-lactone group between C-6a and C-9, was 2-fold more potent than **1**, indicating that the presence of one γ-lactone group in the azafilone analog plays an important role in the antimycobacterial activity. Other compounds isolated from *B. formosana* showing moderate to weak antimycobacterial activity are 5-hydroxy-3,7,4′–trimethoxyflavone (**27**), *N*-*trans*-feruloy-3-*O*-methyldopamine (**29**), methyl-3,4-methylenedioxycinnamate (**30**), 4-methoxycinnamaldehyde (**42**), and 4-methoxy-trans-cinnamic acid (**43**), with MIC values of 25.0, 12.5, 58.2, 42.1, and 50.0 mg/mL, respectively. Meanwhile, 3,4-methylenedioxycinnamic acid (**31**), 3,4-methylenedioxy benzoic acid (**32**), 4-hydroxy benzaldehyde (**39**), 4-(3-methylbut-2-enyloxy)benzoic acid (**41**), and ergosta-4,6,8(14),22-tetraen-3-one (**64**) did not show antimycobacterial activity [10].

### 3.3. Cytotoxic Activity

Natural products obtained from endophytic fungi have been identified as a sustainable and productive source of anticancer agents holding significant potential for the advancement of modern anticancer drugs [86]. Among these, an endophytic fungal strain belonging to *Biscogniauxia* has been identified and reported to produce compounds that are effective in anticancer tests. Some of these compounds include cerebroside A (**3**), cerebroside C (**4**), 7-hydroxy-5-methoxy-4,6-dimethylphthalide (**54**), and 6-[(1*R*)-1-hydroxy-1-methyl-2-propenyl]-4-methoxy-3-methyl-2*H*-pyran-2-one (**56**), nectriapyrone (**59**) [11], biscogniacid A (**82**), biscognienyne A-E (**83**–**86**), dimericbiscognienynes A–C (**87**–**89**) [21,22], biscogniauxiaol A–F (**90**–**95**) [23], pogostol (**103**), xylariterpenoids A–B (**107**,**108**), xylariterpenoids L–N (**109**–**111**), (1*R*,2*S*,6*R*,7*S*)-1,2-dihydroxy-α-bisabolol (**112**) [11]. Subsequently, compounds (**54**), (**103**), (**105**), and (**109**) showed significant toxicity against HeLa cells with an IC_50_ range between 8.64 ± 1.22 and 31.16 ± 4.12 μg/mL [11]. Meanwhile, compounds **90**–**95** showed weak reversal activity against cisplatin-resistant A549/DDP cells [23].

### 3.4. Antioxidants

Endophytic fungi have gained attention as an alternative source of these valuable compounds due to their potential health benefits [87]. Subsequently, *B. capnodes* isolated from the fruit of *Averrhoa carambola* L. (Oxalidaceae), commonly called starfruit, has been found to produce compounds of the isocumarin and dihydroisocoumarin groups. These compounds include 6-*O*-methyl-reticulol (**8**), reticulol (**9**), 7-hydroxy-5-methylmellein (**15**), and 5-methylmellein (**21**) [21]. Among these compounds, reticulol (9) exhibited significant DPPH radical scavenging activity with an IC_50_ value of 58 µg/mL (IC_50_ of the positive control butylated hydroxyanisole was 5.5 µg/mL) [12]. These compounds are believed to have a strong protective mechanism against the generation of free radicals, which cause several disorders such as aging, cancer, atherosclerosis, coronary heart disease, and diabetes. Reticulol exhibits moderate antioxidant activity; this compound was first discovered in a strain of *Streptomyces rubrireliculae* [88]. It has been widely reported to have other biological activities, such as its antitumor properties by deactivating Topo I, which is included in tumor metastasis, and exhibiting excellent cytotoxicity against melanoma B16F10 when combined with adriamycin [89]. Additionally, it serves as an inhibitor of cyclic adenosine 3′,5′-monophosphate phosphodiesterase [79] and cyclic nucleotide phosphodiesterase [90]. Recent research showed its ability to significantly reduce degranulation and histamine release [91].

### 3.5. Antigerminative

The fungus isolated as an endophytic from the plum yew *Cephalotaxus harringtonia*, namely the *B. nummularia* strain, was subjected to a chromatography technique to produce chemical constituents, which were then tested for anti-germinative activity to determine any suspected phytopathogenesis. The anti-germinative tests were carried out using radish seeds, with all compounds tested at a maximum concentration of 100 mg/mL, which is comparable to the effective concentration of glyphosate, a commonly used weed killer constituent. Among the compounds tested, xylaranone (**104**) exhibited the strongest anti-germinative activity with an 85% inhibition rate, surpassing the reference glyphosate (75% inhibition). Xylaranol B also showed significant effectiveness against seed germination, with over 50% inhibition at the tested concentrations. Meanwhile, compounds derived from mellein, namely compounds 10 and 11, had inhibitory effects of less than 50% [13].

### 3.6. Phytotoxic Activity

Phytotoxins are bioactive substances produced naturally by various plants and microbial species (e.g., bacteria and fungi), some of which can be consumed by humans [92]. Fungal phytotoxins, also known as phytotoxic secondary metabolites from fungi, are substances that are naturally produced by fungi through biochemical reactions, and they have toxic effects on plants [93]. Fungal phytotoxins play an important role in the development of plant disease symptoms, including leaf spot, wilting, chlorosis, and necrosis, as well as growth inhibition and enhancement [94]. *Biscogniauxia rosacearum*, first recognized as a pathogen causing grape stem disease in Paveh vineyards (western Iran), produces meso-2,3-butanediol (**115**). (3*R*)-5-methylmellein (**22**), (3*R*)-5-methyl-6-methoxymellein (**23**), tyrosol (**47**). In addition, nectriapyrone (**59**) was produced as a phytotoxin from the same fungal strain isolated from oak trees in the Zagros forest in Gilan-e Gharb, Kermanshah Province. The phytotoxicity of secondary metabolites of *B. rosacearum* was tested by leaf pricking on *Quercus ilex* L. and *Hedera helix* L. and by foliar absorption tests on grapevine (*Vitis vinifera* L.) at concentrations of 5 × 10^−3^ and 10^−3^ M. Among these compounds, **22** and **115** were found to be the most phytotoxic on grapevine. In the case of *Q. ilex*, compounds **46** and **58** induced severe necrosis at the highest concentration, while none of the compounds exhibited activity on *H. helix* [14].

### 3.7. Activity against the Enzyme GSK-3β

The production of bioactive compounds, specifically those with parasitic pathogenic properties or phytotoxic substances, has been extensively examined in various *Bioscogniauxia* strains. One of these strains, *Bioscogniauxia mediterranea* strain LF657, which was isolated from deep-sea sediments of the East Mediterranean Sea at a water depth of 2800 m, produces a new compound identified as the isopyrrolonaphthoquinone group, namely biscogniauxone (**33**) showing activity that inhibits glycogen synthase kinase *GSK-3β* with an IC_50_ value of 8.04 µM (±0.28) [17]. In another investigation, the compound bhimamycin H, the isopyrrolonaphthoquinone group, also inhibited the activity of this enzyme in the same range (IC_50_ value 18 µM) [95]. Therefore, isopyrrolonaphthoquinones and similar structures can be considered potential candidates for drug development to treat diseases associated with *GSK-3β* biological targets, such as type 2 diabetes, neurological disorders, or cancer [96,97,98].

### 3.8. Anti-Acetylcholinesterase (AChE) Activity and Anti-Alzheimer Disease (AD)

AD is the leading cause of dementia, contributing to approximately 75% of all dementia cases. The pathophysiological processes described for the development of AD include neuronal and synaptic degeneration, characterized mainly by cholinergic disturbances. As a result, AChE inhibitors represent the primary class of drugs used in the treatment of the dementia phase of AD [99]. Several investigations have explored the search for natural molecules with AChE inhibitory properties. These investigations have examined various compounds, particularly those falling into categories such as alkaloids, monoterpenes, coumarins, triterpenes, flavonoids, benzenoids, diterpenes, heterocyclic oxygen, sesquiterpenes, stilbenes, lignans, sulfur compounds, proteinids, polycyclics, quinoids, benzoxazines, carotenoids, and alicyclics [100]. Recently, anti-AChE activity of the pthalide group has also been reported, namely from a strain (No. 69-8-7-1) isolated from *Rimelia reticulata*, which was identified as *Biscogniauxia* sp., then extracted and subjected to isolation and purification to produce a new phthalide derivative, biscogniphthalides A–D (compounds **50**–**53**), together with a known compound, [4-[(acetyloxy)methyl]-7-methoxy-6-methyl-1(3*H*)-isobenzofuranone (**49**) [9]. The bioactivity of phthalide was evaluated via an anti-AChE activity test. Results from tests **49**, **50**, and **51** indicated weak inhibition at a concentration of 100 μM when compared to hurperzena-A, used as a positive control, which showed substantial inhibition of 87.66 ± 0.26% [9]. In addition, the anti-AD activities of compounds isolated from Lichen *Usnea Mutabilis* Stirt were also evaluated by the AD fly model, with memantine as the positive control. In this model, transgenic AD flies carry the human Aβ 42 gene, which causes expression of the Aβ 42 peptide in the fly brain and induces AD pathological phenotypes. The results suggest the potential of these compounds as anti-AD drugs [21].

## 4. Conclusions and Future Prospects

Secondary metabolites were produced from *Biscogniauxia*, resulting in the isolation of 115 chemical compounds belonging to the class of secondary metabolites and fatty acids from approximately 9 taxa. The most frequently isolated chemical compounds were in the terpenoid group and the derivatives, with 43 compounds, followed by the coumarin group with 21 compounds. The use of PDA media was the largest amount, namely 75% of the total metabolite compounds produced from *Biscogniauxia*. Secondary metabolites of the *Biscogniauxia* strain have various applications in pharmacology. Many studies were also conducted to confirm associated biological activities, such as antifungal, antimycobacterial, cytotoxic activity, antioxidant, anti-germinative, phytotoxic activity, and the presence of inhibitory activity. GSK-3β enzymes, anti-*AChE* activity, and anti-AD effect the importance of these resources in supporting the discovery of new drugs produced by *Biscogniauxia*, with the potential for further development.

## Figures and Tables

**Table 1:** List of secondary metabolites produced by *Biscogniauxia* collected from the literature.

No.	Compound Name	Fungal Strains (Solvent Used for Extraction)	Formulas	Nominal Mass	Ref.
**Azaphilone Derivatives**					
1.	Biscogniazaphilone A	*B. formosana* (EtOH)	C_24_H_34_O_4_	409.23	[10]
2.	Biscogniazaphilone B	*B. formosana* (EtOH)	C_25_H_32_O_5_	435.21	[10]
**Cerebrosides**					
3.	Cerebroside A	*B. whalleyi* (EtOAc)	C_41_H_75_NO_9_	748.53	[11]
4.	Cerebroside C	*B. whaleyi* (EtOAc)	C_43_H_79_NO_9_	754.1	[11]
**Coumarins**					
**(a)** **Isocoumarins**					
5.	Isoscopoletin	*B. cylindrospora* (EtOH)	C_10_H_8_O_4_	192.17	[4]
6.	Scopoletin	*B. cylindrospora* (EtOH)	C_10_H_8_O_4_	192.04	[4]
7.	Isofraxidine	*B. cylindrospora* (EtOH)	C_11_H_10_O_5_	222.05	[12]
**(b)** **Coumarins**					
8.	6-*O*-methyl-reticulol	*B. capnodes* (EtOAc)	C_12_H_12_O_5_	236.07	[12]
9.	Reticulol	*B. capnodes* (EtOAc)	C_11_H_10_O_5_	222.19	[12]
**(c)** **Dihydroisocoumarin (Melleins)**					
10.	3,5-dimethyl-8-methoxy-3,4-dihydroisocoumarin	*B. nummularia* (EtOAc)	C_11_H_12_O_4_	208.21	[13]
11.	3,5-dimethyl-8-hydroxy-3,4-dihydroisocoumarin	*B. nummularia* (EtOAc)	C_12_H_14_O_3_	206.24	[13]
12.	(3*R*,4*R*)-4-hydroxymellein	*B. rosacearum* (EtOAc)	C_10_H_10_O_4_	194.18	[14]
13.	(3*R*,4*S*)-4- hydroxymellein	*B. rosacearum* (EtOAc)	C_10_H_10_O_4_	194.18	[14]
14.	(3*R*)-6-hydroxymellein	*B. rosacearum* (EtOAc)	C_10_H_10_O_4_	194.18	[14]
15.	7-hydroxy-5-methylmellein	*B. capnodes* (EtOAc)	C_11_H_12_O_4_	208.21	[12]
16.	(3*S*)-5-hydroxy-8-*O*-methylmellein	*B. cylindrospora* (EtOH 70%)	C_11_H_12_O_4_	208.21	[15]
17.	5-hydroxymethylmellein	*B. cylindrospora* (EtOH 70%)	C_11_H_12_O_4_	208.07	[15]
18.	5-formylmellein	*B. cylindrospora* (EtOH 70%)	C_11_H_10_O_4_	206.06	[15]
19.	Mellein-5-carboxylic acid	*B. cylindrospora* (EtOH 70%)	C_11_H_10_O_5_	222.05	[15]
20.	(3*R*)-mellein	*B. rosacearum* (EtOAc)	C_10_H_10_O_3_	178.18	[8]
21.	5-methylmellein	*B. mediterranea* (EtOAc) *B. mediterranea* (DCM) *B. capnodes* (EtOAc) *B. whalleyi* (EtOAc)	C_11_H_12_O_3_	192.21	[16] [16] [12] [11]
22.	(3*R*)-5-methylmellein	*B. rosacearum* (EtOAc)	C_11_H_12_O_3_	193	[14]
23.	(3*R*)-5-methyl-6-methoxymellein	*B. rosacearum* (EtOAc)	C_12_H_14_O_4_	223	[14]
24.	(3*R*)-4-methoxymellein	*B. rosacearum* (EtOAc)	C_11_H_12_O_4_	208.21	[14]
25.	6-methoxy-5-methylmellein	*B. mediterranea* (MeOH)	C_12_H_14_O_4_	222.24	[17]
**Fatty acids**					
26.	Linoleic acid	*B. cylindrospora* (EtOH)	C_18_H_32_O_2_	280.44	[3]
**Flavonoids**					
27.	5-hydroxy-3,7,4′-trimethoxyflavone	*B. formosana* (EtOH)	C_19_H_18_O_7_	358.3	[10]
**Furan**					
28.	(3a*S*,4a*R*,8a*S*,9a*R*)-3a-hydroxy-8a-methyl-3,5-dimethylenedecahydronaphto [2,3-*b*]furan-2(3*H*)-one (HDFO)	*Biscogniauxia* sp. (EtOAc)	C_15_H_20_O_3_	248.31	[18]
**Hydroxycinnamic acids and derivatives**					
29.	*N*-*trans*-feruloy-3-*O*-methyl-dopamine	*B. formosana* (EtOH)	C_19_H_21_NO_5_	343.4	[10]
**Lignans**					
30.	Methyl 3,4-methylenedioxycinnamate	*B. formosana* (EtOH)	C_12_H_14_O_4_	222.24	[10]
31.	3,4-methylenedioxycinnamic acid	*B. formosana* (EtOH)	C_10_H_8_O_4_	192.17	[10]
32.	3,4-methylenedioxybenzoic acid	*B. formosana* (EtOH)	C_8_H_6_O_4_	166.13	[10]
**Napthoquinone**					
33.	Naphtho [2,3-*c*]furandione (isofuranonephthoquinone)	*B. mediterranea* (MeOH)	C_12_H_6_O_3_	198.17	[17]
**Peptides**					
**(a)** **Cyclic dipeptides**					
34.	cyclo (ʟ -Pro-Gly)	*B. whalleyi* (EtOAc)	C_7_H_10_N_2_O_2_	154.16	[11]
35.	cyclo (ʟ-Pro-ʟ-Leu)	*B. whalleyi* (EtOAc)	C_11_H_18_N_2_O_2_	210.27	[11]
36.	cyclo (ʟ-Pro-ʟ-Phe)	*B. whalleyi* (EtOAc)	C_14_H_16_N_2_O_2_	244.29	[11]
37.	cyclo (ʟ-Pro-ʟ-Val)	*B. whalleyi* (EtOAc)	C_10_H_16_N_2_O_2_	196.25	[11]
**(b)** **Cyclopeptide**					
38.	cyclo-(ʟ-Phe-ʟ-Leu-ʟ-Val-ʟ-Leu-ʟ-Leu)	*B. mediterranea* (MeOH)	C_32_H_51_N_5_O_5_	585.8	[17]
**Phenyl and Phenol Derivatives**					
39.	4-hydroxybenzaldehyde	*B. formosana* (EtOH)	C_7_H_6_O_2_	122.12	[10]
40.	5-hydroxy-2-prenylhydroquinone	*B. whalleyi* (EtOAc)	C_11_H_14_O_3_	194.09	[11]
41.	4-(3-methylbut-2-enyloxy)benzoic acid	*B. formosana* (EtOH)	C_13_H_16_O_3_	220.26	[10]
42.	4- methoxycinnamaldehyde	*B. formosana* (EtOH)	C_10_H_10_O_2_	162.18	[10]
43.	4-methoxy-*trans*-cinnamic acid	*B. formosana* (EtOH)	C_10_H_10_O_3_	178.18	[10]
44.	Methylparaben	*B. cylindrospora* (70% EtOH)	C_8_H_8_O_3_	152.15	[15]
45.	Phenylacetic Acid	*B. mediterranea* (EtOAc)	C_8_H_8_O_2_	136.15	[8]
46.	Syringaldehyde	*B. cylindrospora* (EtOH 70%)	C_9_H_10_O_4_	182.17	[15]
47.	Tyrosol	*B. whalleyi* (EtOAc) *B. rosacearum* (EtOAc)	C_9_H_13_NO_2_	167.09	[11,14]
48.	Vanillic acid	*B. cylindrospora* (EtOH 70%)	C_8_H_8_O_4_	168.14	[15]
**Phthalides**					
49.	[4-[(acetyloxy)methyl]-7-methoxy-6-methyl-1(3*H*)-isobenzofuranone	*Biscogniauxia* sp. (EtOAc)	C_13_H_14_O_5_	209.08	[9]
50.	Biscogniphthalides A	*Biscogniauxia* sp. (EtOAc)	C_18_H_23_O_7_	351.14	[9]
51.	Biscogniphthalides B	*Biscogniauxia* sp. (EtOAc)	C_17_H_21_O_7_	337.12	[9]
52.	Biscogniphthalides C	*Biscogniauxia* sp. (EtOAc)	C_12_H_13_O_6_	253.07	[9]
53.	Biscogniphthalides D	*Biscogniauxia* sp. (EtOAc)	C_11_H_13_O_4_	209.08	[9]
54.	7-hydroxy-5-methoxy-4,6-dimethylphthalide	*B. whalleyi* (EtOAc)	C_11_H_12_O_3_	208.07	[11]
**Pyranopyran**					
55.	(*Z*)-2-methoxy-1-[7-((*Z*)-2-methoxybut-2-enoyl)-3,4,5,6-tetramethyl-2*H*,7*H*-pyrano [2,3-*b*]pyran-2-yl]but-2-en-1-one (Biscopyran)	*B. mediterranea* (EtOAc)	C_22_H_28_O_6_	388.18	[8]
**α-pyrones**					
56.	6-(1′, 2′-dimethyloxiran-1′-yl)-4-methoxy-3-methyl-2*H*-pyran-2-one	*B. whalleyi* (EtOAc)	C_11_H_14_O_4_	233.07	[11]
57.	Gulypyrone B	*B. whalleyi* (EtOAc)	C_11_H_14_O_4_	210.09	[11]
58.	6-[(1*R*)-1-hydroxy-1-methyl-2-propenyl]-4-methoxy-3-methyl-2*H*-pyran-2-one	*B. whalleyi* (EtOAc)	C_11_H_14_O_4_	210.23	[11]
59.	Nectriapyrone	*B. whalleyi* (EtOAc) *B. rosacearum* (EtOAc)	C_11_H_14_O_3_	194.23	[11] [14]
60.	Phomopyrone A	*B. whalleyi* (EtOAc)	C_11_H_14_O_4_	210.22	[11]
61.	Vermopyrone	*B. whalleyi* (EtOAc)	C_9_H_10_O_4_	182.17	[11]
**Steroids**					
62.	Cerevisterol	*B. whalleyi* (EtOAc)	C_28_H_46_O_3_	430.34	[11]
63.	Ergone	*B. whalleyi* (EtOAc)	C_28_H_40_O	392.6	[11]
64.	Ergosta-4,6,8(14), 22-tetraen-3-one	*B. formosana* (EtOH)	C_28_H_40_O	392.6	[10]
65.	Ergosterol	*B. whalleyi* (EtOAc)	C_28_H_44_O	396.34	[11]
66.	Ergosterol peroxide	*B. whalleyi* (EtOAc)	C_28_H_44_O_3_	428.6	[11]
67.	3β-hydroxystigmast-5-en-7-one	*B. cylindrospora* (EtOH 70%)	C_29_H_48_O_2_	428.7	[15]
68.	β-cytostenone	*B. cylindrospora* (EtOH)	C_29_H_48_O	412.7	[4]
69.	β-sitosterol	*B. cylindrospora* (EtOH)	C_29_H_50_O	414.7	[4]
**Terpenoids and the Derivatives**					
**(a)** **Diterpenoids**					
70.	Biscognisecoisopimarate A	*Biscogniauxia* sp. (EtOAc)	C_23_H_38_O_6_	433.25	[19]
71.	3β-Hydroxyrickitin A	*Biscogniauxia* sp. (EtOAc)	C_20_H_27_O_4_	331.19	[19]
**(b)** **Meroterpenoids**					
72.	Biscogniacid B	*Biscogniauxia* sp. (EtOAc)	C_12_H_15_O_5_	239.09	[20]
73.	Biscogniacid C	*Biscogniauxia* sp. (EtOAc)	C_12_H_15_O_5_	239.09	[20]
74.	Biscognienyne D	*Biscogniauxia* sp. (EtOAc)	C_16_H_20_O_4_	299.12	[20]
75.	Biscognienyne F	*Biscogniauxia* sp. (EtOAc)	C_17_H_20_O_6_	343.11	[20]
76.	Biscognin A	*Biscogniauxia* sp. (EtOAc)	C_16_H_23_O_5_	295.15	[20]
77.	Biscognin B	*Biscogniauxia* sp. (EtOAc)	C_1 6_H_23_O_4_	279.16	[20]
78.	Biscognin C	*Biscogniauxia* sp. (EtOAc)	C_15_H_21_O_4_	265.14	[20]
79.	Biscognin D	*Biscogniauxia* sp. (EtOAc)	C_16_H_21_O_4_	277.14	[20]
80.	Biscognin E	*Biscogniauxia* sp. (EtOAc)	C_16_H_21_O_4_	277.14	[20]
81.	Biscognin F	*Biscogniauxia* sp. (EtOAc)	C_16_H_22_O_4_	301.214	[20]
82.	Biscogniacid A	*Biscogniauxia* sp. (EtOAc)	C_12_H_13_O_4_	221.08	[21]
83.	Biscognienyne A	*Biscogniauxia* sp. (EtOAc)	C_16_H_22_O_3_	285.14	[21]
84.	Biscognienyne B	*Biscogniauxia* sp. (EtOAc)	C_16_H_20_O_3_	283.13	[21]
85.	Biscognienyne C	*Biscogniauxia* sp. (EtOAc)	C_15_H_20_O_4_	287.12	[21]
86.	Biscognienyne E	*Biscogniauxia* sp. (EtOAc)	C_16_H_20_O_4_	283.13	[20]
87.	Dimericbiscognienynes A	*Biscogniauxia* sp. (EtOAc)	C_32_H_40_O_6_	545.28	[21]
88.	Dimericbiscognienynes B	*Biscogniauxia* sp. (EtOAc)	C_32_H_42_O_6_	545.28	[22]
89.	Dimericbiscognienynes C	*Biscogniauxia* sp. (EtOAc)	C_32_H_41_O_6_	521.29	[22]
**(c)** **Sesquiterpenoids**					
90.	Biscogniauxiaol A	*B. petrensis* (MeOH)	C_15_H_23_O_3_	251.1644	[23]
91.	Biscogniauxiaol B	*B. petrensis* (MeOH)	C_15_H_28_O_3_	279.1927	[23]
92.	Biscogniauxiaol C	*B. petrensis* (MeOH)	C_15_H_28_O_4_	295.1882	[23]
93.	Biscogniauxiaol D	*B. petrensis* (MeOH)	C_15_H_28_O_4_	295.187	[23]
94.	Biscogniauxiaol E	*B. petrensis* (MeOH)	C_15_H_28_O_4_	295.1878	[23]
95.	Biscogniauxiaol F	*B. petrensis* (MeOH)	C_16_H_28_O_4_	307.1873	[23]
96.	Biscogniauxiaol G	*B. petrensis* (MeOH)	C_15_H_28_O_3_	277.1764	[23]
97.	Epiguaidiol A	*B. whalleyi* (EtOAc)	C_15_H_26_O_2_	238.37	[11]
98.	Graphostromane E	*B. whalleyi* (EtOAc)	C_15_H_26_O_2_	261.18	[11]
99.	(1R*,4S*,5S*,7S*,10R*)-guaia-11(12)-en-7,10-diol	*B. whalleyi* (EtOAc)	C_15_H_26_O_2_	261.18	[11]
100.	(1*R*,4*S*,5*S*,7*R*,10*R*,11*R*)-guaiane-10,11,12-triol	*B. whalleyi* (EtOAc)	C_15_H_27_O_3_	255.37	[11]
101.	(1*R*,4*S*,5*S*,7*R*,10*R*,11*S*)-guaiane-10,11,12-triol	*B. whalleyi* (EtOAc)	C_15_H_27_O_3_	255.37	[11]
102.	Patchouliguaiol A	*B. whalleyi* (EtOAc)	C_15_H_24_O_2_	236.34	[11]
103.	Pogostol	*B. whalleyi* (EtOAc)	C_15_H_26_O	222.37	[11]
104.	Xyralanone	*B. whalleyi* (EtOAc) *B. nummularia* (EtOAc)	C_15_H_27_O_3_	255.37	[11,13]
105.	Xylaranol A	*B. whalleyi* (EtOAc)	C_8_H_10_O_4_	170.16	[11]
106.	Xylaranol B	*B. nummularia* (EtOAc)	C_15_H_28_O_3_	257.29	[13]
107.	Xylariterpenoids A	*B. whalleyi* (EtOAc)	C_15_H_25_O_3_	253.36	[11]
108.	Xylariterpenoids B	*B. whalleyi* (EtOAc)	C_15_H_25_O_3_	253.36	[11]
109.	Xylariterpenoid L	*B. whalleyi* (EtOAc)	C_15_H_22_O_2_	257.15	[11]
110.	Xylariterpenoid M	*B. whalleyi* (EtOAc)	C_15_H_24_O_4_	291.15	[11]
111.	Xylariterpenoid N	*B. whalleyi* (EtOAc)	C_15_H_23_O	219.17	[11]
112.	(1*R*,2*S*,6*R*,7*S*)-1,2-dihydroxy-α-bisabolol	*B. whalleyi* (EtOAc)	C_15_H_22_O	277.17	[11]
**Tyramines**					
113.	*N*-*trans*-feruloyltyramine	*B. cylindrospora* (EtOH 70%)	C_18_H_19_NO_4_	313.35	[15]
114.	*N*-*cis*-feruloyltyramine	*B. cylindrospora* (EtOH 70%)	C_18_H_19_NO_4_	313.3	[15]
**Other**					
115.	Meso-2,3-butanediol	*B. rosacearum* (EtOAc)	C_4_H_10_O_2_	90.07	[14]

**Table 2:** Occurrence of secondary metabolites in *Biscogniauxia* strain and biological activities studied.

Strains	The Part Where the Mushroom Grows	Growing Conditions	Identified Compounds	Biological Activity	Ref.
*B. mediterranea* Ohu 19B	*Opuntia humifusa* plant	Potato dextrose agar (PDA) medium	20	Antifungal *(C. fragariae, C. gloeosporioide C. acutatum)*	[16]
*B. petrensis* MFLUCC14-0151	*Dendrobium orchids*	Martin modified (MM) medium	90–96	Antifungal (*C. albicans*)	[23]
*B. formosana* BCRC 33718	*Cinnamomum* sp	Potato dextrose agar (PDA) medium	1, 2, 26, 28–31, 38, 40–42, 63	Antimycobacterial (*M. tuberculosis*)	[10]
*B. whalleyi* SWUF13-085	*Corticated* wood	Potato dextrose agar (PDA) medium	3, 4, 53, 57, 58, 82–90, 107–112	Cytotoxic activity (HeLa cells, HT29, HT116 cells, MCF-7 cells, Vero cells) and NO production inhibition	[11]
*B. capnodes* TAC-2014	*Averrhoa carambola*	Potato dextrose broth (PDB) medium	7, 8, 15, 21	Antioxidant activity	[12]
*B. rosacearum* IRAN 4194C and IRAN 4287C	*Vitis vinifera* L.	Potato dextrose broth (PDB) medium	12–14, 20, 22–24, 46, 58, 115	Phytotoxic activity	[14]
*B. nummularia* LCP 05669	*Cephalotaxus harringtonia*	Potato dextrose agar containing V8 medium	10, 11, 104, 106	Antigerminative activity	[13]
*B. mediterranea* LF657	Deep-sea sediments	Five different agar media (Cytophaga-Flavobacterium-Bacteroides medium, tryptone, yeast extract, Bacto™ agar)	24, 32, 37	Activity against the enzyme GSK-3β	[17]
*Biscogniauxia* sp. No. 71-10-1-1	*Usnea mutabilis* Stirt.	-	82–95, 87 82, 87	Cytotoxic activity (HeLa, SW480, PANC-1) Anti-AD activities	[21]
*Biscogniauxia* sp. No. 69-8-7-1	*Rimelia reticulata*	Potato dextrose agar (PDA) medium	49–52	AChE activity	[9]

## Data Availability

This research did not report any data.
